# The impact of insufficient sleep on the serial reproduction of information

**DOI:** 10.1093/sleepadvances/zpaf026

**Published:** 2025-05-04

**Authors:** David L Dickinson, Sean P A Drummond

**Affiliations:** Economics and CERPA, Appalachian State University, Boone, NC, United States; Economic Science Institute, Chapman University, Orange, CA, United States; Institute of Labor Economics, IZA, Bonn, Germany; School of Psychological Sciences, Monash University, Clayton, VIC, Australia

**Keywords:** sleep restriction, cognition, communication, information transmission

## Abstract

Story retelling is an important form of communication, cultural practice, and message transmission. Insufficient sleep is known to affect relevant cognitive skill areas necessary for story retelling or transmission fidelity. We conducted a preregistered randomized cross-over study on *n* = 155 young adults with exogenously assigned nightly sleep levels experienced in their at-home environments. A serial story reproduction task was administered online, and chains of up to three retells of a given story involved varied numbers of sleep restricted (SR) versus well-rested (WR) retellers. While story content decayed with each retell, group-level analysis showed that additional SR retellers in a chain was associated with greater decay, which mostly resulted from the introduction of an initial SR reteller at the first retell. Supporting the group-level effect, individual-level analysis confirmed that the number of details and the story’s key event were significantly less preserved during a participant’s SR treatment week. Exploratory analysis showed an attenuation of this effect in those reporting a higher level of affective response (interest or surprise) in the story. This suggests that emotional engagement can combat the deleterious effects of SR on successful story retelling, and perhaps on other types of content recollection.

**Statement of Significance:**

Sleep restriction can negatively impact one’s ability to faithfully reproduce (i.e. retell) a short story. In our study, participants retold multiple stories in both a well-rested and sleep restricted state, and some retells were of stories that had previously been retold. Story content decayed with retelling, which we document by examining the number of characters, details, and preservation of the story’s key event, relative to the source story viewed by the participant. Sleep restriction significantly increased content decay within a retell. Interestingly, exploratory analysis found that higher self-reported levels of “surprise” or “interest” in the story mitigated the negative sleep-restriction effect. This result highlights the role of affective engagement as a countermeasure to sleep restriction in facilitating information reproduction.

Communication takes on various forms, and the ability to accurately retell a story or other information has relevance in many different arenas (e.g. workplace, social networks, family communications, etc.). The relay of information or message content has important safety implications in some occupational settings, such as emergency services, medical settings, or air traffic control [1]. In other instances, story retelling can have legal implications, such as with witness testimony [2]. In educational settings retelling is considered a generative learning strategy [3] and, though limitations exist, and its efficacy has not yet been validated, retells are commonly used for reading comprehension assessments for K-12 education [4, 5]. Finally, story retelling is an important means to transmit cultural knowledge in many parts of the world [6]. A systematic study of factors that influence the accuracy of content transmission can therefore offer insights into a wide range of environments where communication reliability matters.

Insufficient sleep is commonplace in modern societies [7–9], and our study contributes by examining how commonplace levels of insufficient sleep affect retell fidelity in a serial story reproduction task. Information transmission is not the only relevant aspect of communication [10]. Conveying the emotional appraisal of a narrative is important because the affective valence of words can be remembered better than actual words, as noted in a telephone game serial reproduction study [11]. Breithaupt et al. [12] further suggest that emotional appraisals in story retelling can be an anchor to help remember story content, perhaps especially in narrative communication. We therefore also contribute exploratory analysis examining whether affective engagement moderates how one’s sleep state affects retell fidelity.

A foundational theoretical proposition in communications research is that communication fidelity is enhanced when both the source and recipient have sufficient physiological and cognitive processing skills [13]. This implicates any temporary state of impaired cognition, such as sleepiness, as problematic as it pertains to message relay and communication fidelity. The known effects of sleep deprivation on cognition [14] as well as on cognitive function relevant to communication fidelity (e.g. verbal working memory and verbal learning [15–17]), suggest that sleep loss is likely to magnify any breakdown in message transmission reliability, though research has been limited in this area.^1^ To our knowledge, few studies have examined the effects of sleep deprivation on communication (e.g. [22, 23]), and their focus has been on interpersonal verbal communication of the sort where numerous cognitive domains are involved (e.g. verbal fluency and perception, linguistic comprehension, audio processing). A related paper recently examined the impact of sleep on story retrieval and memory distortion, using an overnight sleep versus daytime wake protocol with a 12 hours delayed recall. The authors reported: (1) sleep helped memory retrieval of a story after one had only listened to the story but had not actively practiced retrieval prior to sleep and (2) sleep led to greater insertion of false memory into the recall [24]. Our sleep protocol differs substantially in that we examined a mixed within/between subjects design with a sleep restriction manipulation in comparison to a well-rested sleep levels condition, and the story recollection was elicited immediately after visual presentation. In the context of our controlled, within-subjects, sleep manipulation protocol, our story retell paradigm is unique in its focus on serial information recall/transmission that does not involve face-to-face communication, social interaction, or audio processing.

## Materials and Methods

The sleep protocol was preregistered on the Open Science Framework (OSF) (https://doi.org/10.17605/OSF.IO/NSPRK) and the telephone task study was separately preregistered (https://doi.org/10.17605/OSF.IO/H4Z6N) to include study design, hypotheses, sample size, and analysis plans.

### The sleep protocol

Participants were screened to meet our inclusion criteria: young adult (18–40 years of age), non-extreme diurnal preference type (using the reduced morningness-eveningness scale [25]): no self-reported diagnosed sleep disorder (or suspected disorder), no self-reported dietary restrictions, and not at significant risk of major depressive (using the Patient Health Questionnaire-2 scale [26]), or anxiety (using the Generalized Anxiety Disorder scale [27]) disorders. Most participants were college students at a mid-sized regional university.^2^

Eligible participants were randomly assigned (prior to recruitment) to “treatment” or “control” conditions for a 3-week study, where Weeks 1 and 3 for treatment participants involved exogenously assigned well-rested (WR: 8–9 hours/night) and sleep-restricted (SR: 5–6 hours/night) sleep levels in randomly assigned order—control participant sleep levels were WR for both Weeks 1 and 3, and Week 2 was a washout week for all participants. Eligible participants were sent a detailed email invitation to participate in the 3-week study only after random assignment to condition and treatment order. The final number of completed control participants (*n* = 37) was notably smaller than the final number of completed treatment participants (*n* = 118), and this was part of the preregistered plan for our study. The intention of the control condition was to document a lack of repeat measures effects (i.e. practice effects) on other cognitive tasks administered twice (in identical form) in the larger project—though that is less of a concern here given the repeat administration of our story retelling task utilized unique stories across administrations. The control condition was also intended to help further validate the treatment condition by documenting the lack of significant within-subject difference in nightly total sleep time across two distinct WR condition weeks (see footnote 7). These control participants were administered the story retelling task just as the treatment participants, except that they were in a WR state at each administration, and so they are nonetheless valuable in increasing the statistical power of our data set. We therefore included the control participants in the current analyses to help increase sample sizes of the WR-condition story retells in the task, and also because their story retells were used as source stories for other study participants. Regarding the protocol’s assigned sleep levels, sleep occurred in one’s home environment as opposed to a sleep lab, was verified by wrist-worn actigraphy, and participants were allowed free choice of their bed and wake times with the treatment condition focused on the amount of sleep opportunity (i.e. time-in-bed) and not its timing. Participants attended lab sessions at the end of treatment Weeks 1 and 3 to participate in other in-lab tasks unrelated to the Telephone Game task.

Overall, *n* = 137 and *n* = 39 participants were initially enrolled in the treatment and control groups, respectively.^3^ Of those enrolled in the treatment condition, 7 failed to show for the initial study session and 12 did not finish the protocol, resulting in the final sample of *n* = 118 treatment participants. Of those enrolled in the control condition, 2 failed to show for the initial session, resulting in the final *n* = 37 sample of control participants. The telephone game task was a required part of the protocol and was administered via an online survey sent to participants after the 6th night of each prescribed-sleep protocol week (i.e. Weeks 1 and 3). As such, participants had experienced a full 6 nights of prescribed sleep levels prior to the telephone game task.

### The telephone game task

The original set of three stories [11] was supplemented with three additional stories we constructed, and pilot tested for similarity. Stories were similar in number of characters, details, and the fact that a key event (detail) was presented that resolved the main character’s core problem within that story. Summary results in the Appendix (Figure A1) show that the newly generated Amanda (A), Jessica (Je), and Eric (E) stories compared favorably on most dimensions with the original Robert (R), Sarah (S), and Jason (Ja) stories. Because a story’s key event was preserved at a somewhat higher rate in the newly constructed stories, we generated two sets of three-stories for our repeated-measures study by mixing and matching original and new stories—we created sets of Robert-Erica-Jessica (R-E-Je) and Jason-Amanda-Sarah (Ja-A-S). The ordering of the story blocks R-E-Je versus Ja-A-S was varied across participants both within and across participant cohorts, and each participant retold each of the six stories exactly once (three stories in randomized order after each sleep treatment week; see Appendix A for details). After each retell, we elicited participants’ self-reported affective ratings on a 1-9 Likert scale for *Surprise* (how surprised you were in the story) and *Interest* (how interested you were in the story). As a methodological choice, we did not incentivize the online task (i.e. study compensation did not vary based on accuracy of story retelling). The concern was a lack of experimental control over possibly copying (e.g. screenshot or photo) the story to ensure retell accuracy.^4^

**Figure 1.**
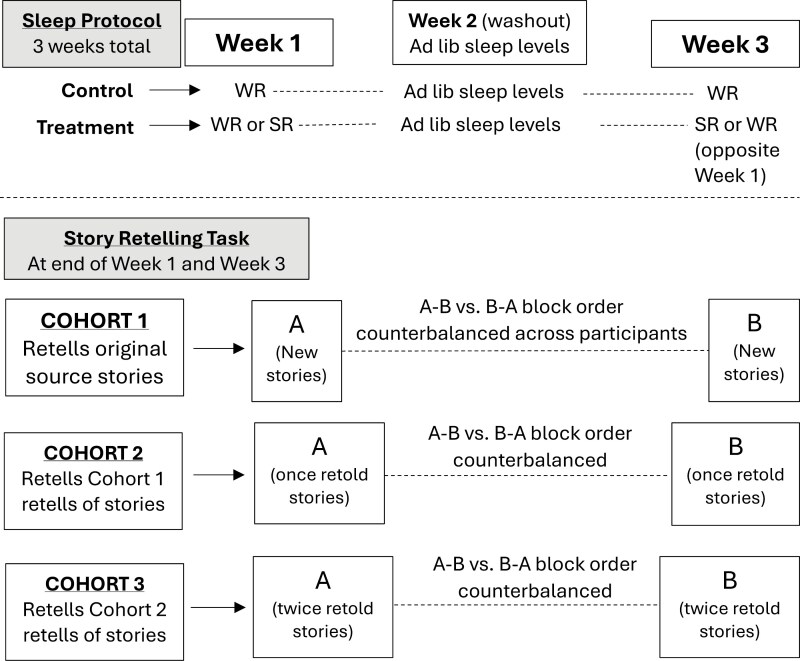
Protocol and procedure for each 3-Cohort set of task story retell. Sleep treatment order (SR-WR vs WR-SR) was counterbalanced across participants. Story Block A = Robert, Eric, Jessica stories. Story Block B = Jason, Amanda, Sarah stories. Story order was randomized within the story block. For Cohorts who retold a previous participant’s retell (Cohorts 2 and 3), all retells from the previous cohort were included within the survey set, and retold stories were randomly drawn in balanced fashion to present to next participant (i.e. each retell was drawn once before the possibility of drawing the same retold story, which only occurred when there were more participants in the latter cohort than in the previous cohort generating the retells). Therefore, each participant in the latter cohort saw one randomly drawn retell of each story from the prior cohort. The retellings of once-retold stories by Cohort 2 are the “Two Retells” data, while the retellings of twice-retold stories for Cohort 3 are the “Three Retells” data in our design. Procedure was similar for administration of the task to Cohorts 4–6, and again for Cohorts 7–9.

To facilitate the creation of 1st, 2nd, and 3rd retellings for the main study, we organized the design using sets of 3 cohorts, with each cohort comprised of 8–16 participants. For example, Cohort 1 would retell the original source stories. These retellings were then used as source stories for Cohort 2 participant retells (i.e. 2nd retellings). Those 2nd retellings were then source stories for Cohort 3 to create a set of 3rd retellings. This process was used for study Cohorts 1–3, Cohorts 4–6, and Cohorts 7–9. Retold stories were randomized in their presentation to new participants. Figure 1 visualizes both the sleep protocol and the procedures for story retelling assignments for each set of 3 cohorts of participants. Data collection was completed after 11 cohorts, and so adjustments to the procedures were made for the final two cohorts for a couple of reasons: First, it was clear that our sample size needs and available budget would dictate only needing two additional cohorts (not another set of three cohorts). Secondly, we made efforts to increase the sample sizes of relatively more rare instances of all-WR or all-SR participants in the retell chain (especially for 2 and 3 retells) for these final cohorts 10 and 11.

Two research assistants independently scored each story for characters, details, and event preservation using the same rubric created for pilot data analysis.^5^ One of the experimenters independently scored the retell in the event the research assistants’ scoring differed by more than 5 details or if there was any disagreement in the key event preservation scoring—*Event Preservation* was coded as 0,.5, or 1, (*Event Preservation = *.5 represented that the key event was preserved “in part”).

### *Preregistered hypotheses* ^6^

Our hypotheses were derived from existing empirical evidence cited previously. Hypothesis H1b below is a replication test of a key affective response result previously reported [11], which suggested that emotional appraisals of a story are resilient even when content fidelity declines. We note that hypotheses H1a and H1b were general and not made conditional on sleep condition or group assignment.

H1a: Characters, number of details, and story event preservation will decline with each retelling of a story.

H1b: Average surprise ratings of a story will stay the same across retellings.

H2: Across multiple retellings of a story, the decline in characters, details, and key event presence will be more significant when more SR participants are in the retell chain.

H3–H5: Characters (H3), Details (H4), and Key Event Preservation (H5) will decline more when the story is retold by an SR, compared with WR, participant.

### Statistical methods

Treatment (SR vs WR) and group-level differences examining the number of SR participants in a retell chain were tested using two-sample *t*-tests. We pooled the control and treatment participant data for the analysis to increase the sample size and statistical power of the analysis, given the lack of significant participants differences between relevant measures such as sex, age, and WR retell characteristics (*p > *.05 in each instance for the *t*-test comparison between treatment participants in the WR condition and control participants—two-sample proportions test used to examine sex differences, which were also insignificant, *p > *.10) However, WR participants had significantly higher levels of nightly sleep (averaging their two WR sleep weeks) compared with treatment participants during the WR condition (*p < *.01, see also Table 1 measures). Thus, while the groups are pooled in our analysis, we conducted sensitivity analysis that using actigraphy-measures nightly sleep in place of the binary SR indicator to account for sleep differences within a condition. These sensitivity analyses are shown in Appendix B, and they support the same conclusions we reach in our main analysis that controls for the participant’s sleep condition with the binary SR indicator variable.

**Table 1.** Mean values of nightly sleep and story content decay (by sex)

Condition	Sex	Nightly sleep (minutes)	CHARACTER content decay	DETAILS content decay	EVENT PRESERVATION content decay
Control (WR week 1)	Female	482.054	−0.2348	−0.2150	−0.1654
	Male	470.435	−0.2945	−0.2417	−0.2000
Control (WR week 3)	Female	474.448	−0.2406	−0.1832	−0.1449
	Male	461.454	−0.2768	−0.2275	−0.2938
Treatment (SR week)	Female	327.249	−0.2390	−0.2090	−0.1492
	Male	317.881	−0.3129	−0.3037	−0.2337
Treatment (WR week)	Female	457.015	−0.2288	−0.2010	−0.0358
	Male	425.570	−0.2831	−0.2885	−0.1700

Story content mean values are averaged across all stories retold by the participant, and the decay is *relative* to the content of the source story the participant viewed for that retell.

Individual-level analysis used multivariate random-effects generalized least squares estimation with robust standard errors clustered on the participant. Sensitivity analysis that involved a sample-selection correction used a weighted least squares regression that accounted for the probability of finishing the protocol (i.e. study attrition), conditional on enrollment (selection equation estimation results that derived these probabilities are given in Appendix B, Table B5). Significance was evaluated using the one-tailed test appropriate for any preregistered directional hypothesis (otherwise, two-tailed significance is reported). Note that while there is an inherent ceiling effect on story content as it is continually retold—retelling a story that was already retold with a 25% loss in its key details means full original story details cannot possibly be achieved—our approach to the analysis mitigates this concern in the sense that we examine the accuracy of a participant’s retell with respect to the story content he/she received. Thus, a participant who retells a story that had lost details or the key event is not penalized except with respect to the version of the story presented to the participant.

## Results

A total of *n* = 155 participants (*n* = 118 treatment, *n* = 37 control) completed the study. The exogenous sleep assignment affected both objective and subjective sleepiness measures across treatment weeks as anticipated; during SR compared with WR, the participants had lower (objective) actigraphy-measured nightly sleep (*p* < .001), higher self-reported sleepiness (*p < *.001), lower perceived cognitive functioning (*p* < .001), lower positive mood (*p* < .001), higher negative mood (*p* < .001), and a self-assessed reduced sleep compared with typically (*p* < .001).^7^Table 1 shows the mean values of nightly sleep (in minutes), as well as mean values of the key story content retention/decay values relative to the source story one viewed, which we break down by sex, treatment, and condition week.

Outcomes were assessed at the level of a chain of up to three retells and at the individual retell level. Hypotheses 1a and 1b are replication tests of main effects of retells fidelity and affective assessment of the story [11]. Table 2 shows results from regressions predicting the percent decline in characters, details, and key event preservation. Indicators for 2nd and 3rd retells are the key coefficient estimates of interest, along with the constant term that describes the level of content decay during the average 1st retell of a story. Tests at the bottom of Table 2 highlight support for Hypothesis 1a by showing all content displays significant decay at each retell—the positive coefficients on the Retell2 and Retell3 indicators indicate the fidelity is lost at a lesser rate in subsequent retells compared with the 1st retell. Hypothesis 1b is tested by examining self-reported *Surprise* and *Interest* ratings after each retell, and here, the data show mixed support for H1b. Specifically, *Surprise* and *Interest* ratings decline to a small extent upon the 3rd retell, compared with the 2nd retell, though they are not different from initial affective ratings at the 1st retell (Table 2). Also, average *Surprise* and *Interest* ratings are lower during SR (*p < *.001 in both instances: two-sample *t*-test), and the decline in *Surprise* upon 3rd tell is greater during SR (again, see tests on the relevant linear coefficient combination at bottom of Table 3).

**Table 2.** The impact of retelling on story reproduction

	(1)	(2)	(3)
VARIABLES	% Characters Lost	% Details Lost	% Event Preservation Lost
			
Retell2 (=1)	0.06	0.07*	0.03
	(0.03)	(0.03)	(0.05)
Retell3 (=1)	0.13**	0.08**	0.12**
	(0.03)	(0.02)	(0.04)
Age	0.01**	0.01**	0.00
	(0.00)	(0.00)	(0.01)
Female (=1)	0.08**	0.10**	0.09*
	(0.03)	(0.02)	(0.04)
Minority (=1)	0.02	0.02	0.06
	(0.03)	(0.02)	(0.06)
MEQ	0.01**	0.01*	0.00
	(0.00)	(0.00)	(0.01)
CRTscore	0.02**	0.02**	0.02
	(0.01)	(0.01)	(0.01)
Repeat Administration (=1)	0.01	0.01	-0.04
	(0.01)	(0.01)	(0.04)
Constant	−0.83**	−0.71**	−0.38*
	(0.10)	(0.08)	(0.15)
Observations	924	924	851
# Participants	155	155	155
Story Fixed Effects	YES	YES	YES
R-squared	0.185	0.154	0.0276
** Tests of decay by retell #**			
1st Retell test: H_0_: Z-test Constant term = 0	−8.33**	−8.91**	−2.53**
2nd Retell test: H_0_: *Χ*^2^ test Constant + Retell2 = 0	65.10**	69.26**	5.01*
3rd Retell test: H_0_:*Χ*^2^ test Constant + Retell3 = 0	48.84**	66.33**	2.93*

**p < .01, *p < .05 for the one-tailed preregistered directional test (otherwise, two-tailed tests). Robust standard errors in parentheses.

**Table 3.** Surprise and Interest across retells

	(1)	(2)	(3)	(4)
VARIABLES	DV= Surprise	DV= Interest	DV= Surprise	DV= Interest
				
SR (=1)	−0.50**	−0.44**	−0.04	−0.29
	(0.15)	(0.13)	(0.28)	(0.27)
Retell2 (=1)	0.22	0.37	0.36	0.46
	(0.28)	(0.27)	(0.32)	(0.31)
Retell3 (=1)	−0.50*	−0.38	−0.24	−0.32
	(0.25)	(0.24)	(0.28)	(0.28)
Retell2 * SR	—	—	−0.44	−0.26
			(0.38)	(0.35)
Retell3 * SR	—	—	−0.72*	−0.17
			(0.36)	(0.34)
Age	−0.00	0.01	−0.00	0.01
	(0.05)	(0.04)	(0.05)	(0.05)
Female (=1)	0.42	0.50*	0.43	0.50*
	(0.23)	(0.23)	(0.22)	(0.23)
Minority (=1)	0.40	0.87**	0.41	0.87**
	(0.30)	(0.30)	(0.30)	(0.30)
MEQ	0.03	0.02	0.03	0.02
	(0.03)	(0.03)	(0.03)	(0.03)
CRTscore	0.07	-0.04	0.07	-0.04
	(0.06)	(0.06)	(0.06)	(0.06)
Repeat Admin (=1)	−0.32*	−0.48**	−0.31*	−0.47**
	(0.13)	(0.12)	(0.13)	(0.12)
Constant	4.42**	3.72**	4.27**	3.65**
	(1.09)	(1.03)	(1.13)	(1.04)
Observations	929	929	929	929
# Participants	155	155	155	155
Story Fixed Effects	YES	YES	YES	YES
R-squared	0.108	0.121	0.114	0.120
*Χ* ^2^ Test of Retell2 = Retell3	7.44**	7.89**	4.16*	6.60**
*Χ* ^2^ Test of Retell2 = Retell2*SR = 0	—	—	0.04	0.35
*Χ* ^2^ Test of Retell3 + Retell3*SR = 0	—	—	8.55**	2.47

**p < .01, *p < .05 for the one-tailed preregistered directional test (otherwise, two-tailed tests). Robust standard errors in parentheses.

**Figure 2.**
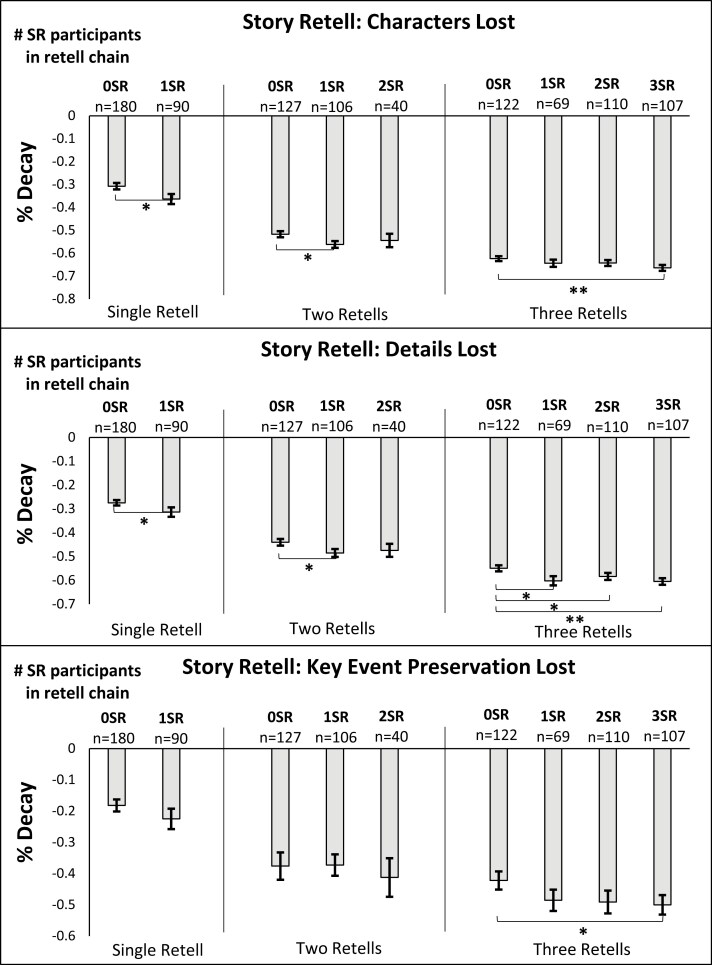
Serial reproduction chain outcomes—the influence of SR. *p < .05, **p < .01 for the one-tailed (preregistered) two-sample *t*-test comparisons. #SR refers to the number of SR participants out of the total who were in the chain of retells (e.g. 0SR and 3SR in the “Three Retells” panel indicates *only* WR (no SR) and *only* SR (no WR) participants, respectively, retold the story in the chain of retells). The number of observations is listed above each bar, which refers to the number of unique story retells contributing to that bar but pooling across the different stories.

Hypothesis 2 is a test that content will decay more with more SR retells in the chain. Figure 2 shows cumulative % of lost content by the length of the retell chain and by composition of SR/WR retells in the chain—here, the additional decay for longer chains of retells further supports Hypothesis 1a. We test Hypothesis 2, using two-sample *t*-tests, by pooling data from all retells resulting from a specific number of SR participants in the chain. Support for Hypothesis 2 is somewhat mixed in this group-level analysis. The two upper panels in Figure 2 show some evidence that more SR retells significantly reduces preservation of *Characters* and *Details*. However, the data on chains of two- and three-retells show that the additional SR reteller does not necessarily worsen the *Characters* or *Details* decay at the margin. And, when examining *Event Preservation*, the only statistically significant decay due to SR retells is found in the three-retell data that compared *Event Preservation* of a story retold three times by all SR compared with all WR participants.

We next examine Hypotheses 3–5 using individual-level analysis. Here, our data are structured as a panel data set with six observations per participant.^8^ We estimated models to predict the story content retained—greater content retention is equivalent to lesser content decay. Specifically, a participant who completed a 2nd or 3rd retell of the story has her outcomes compared with the content present (i.e. preserved) in the retold version of the story she saw. Regression models were estimated using the binary SR indicator as the key sleep measure, with several other specifications used to perform sensitivity analysis. Sensitivity analysis included using continuous average total nightly sleep time (actigraphy measured) as an alternative control for sleep level, and a weighted regression approach to account for possible sample-selection (the inverse probability weight, or IPW, correction specification).^9^ Finally, we estimated models with and without additional covariates to control for demographics, a cognitive reflection measure (CRT score), a repeat administration control for Week 3 versus Week 1 retells, and story fixed effects. We refer to the specifications with or without additional covariate controls in the model as “controls” versus “simple” specifications, respectively.

**Figure 3.**
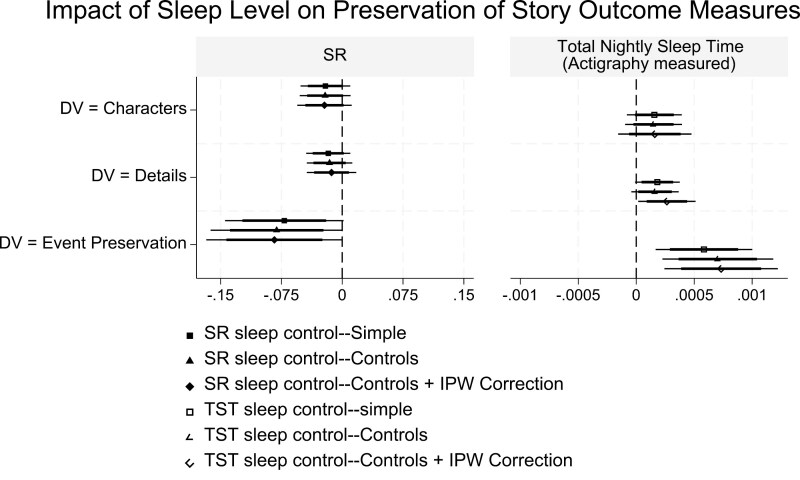
Individual-level retell outcomes—the influence of SR. Plot shows the coefficient estimate with the 99% (thin line) and 95% (thick line) one-tail test confidence interval on the preregistered hypothesis. Full estimation results are in Appendix B, Tables B2–B4.

The results are summarized via coefficient plots in Figure 3, with full results in Appendix B, Tables B1–B3. We report no significant impact of sleep (SR or total nightly sleep time) on *Characters* preserved in a retell, but less total nightly sleep is estimated to significantly reduce *Details* preserved (*p < *.05). We find robust evidence that SR (or lower levels of nightly sleep) significantly lowers *Event Preservation*. Overall, these findings support the hypothesis of a greater fidelity decay when the story is retold by a sleep restricted participant.

### Exploratory analysis—affective engagement in story

**Figure 4.**
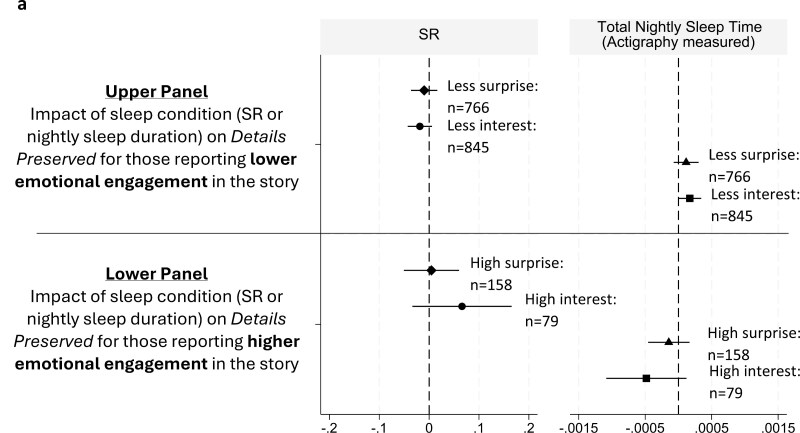

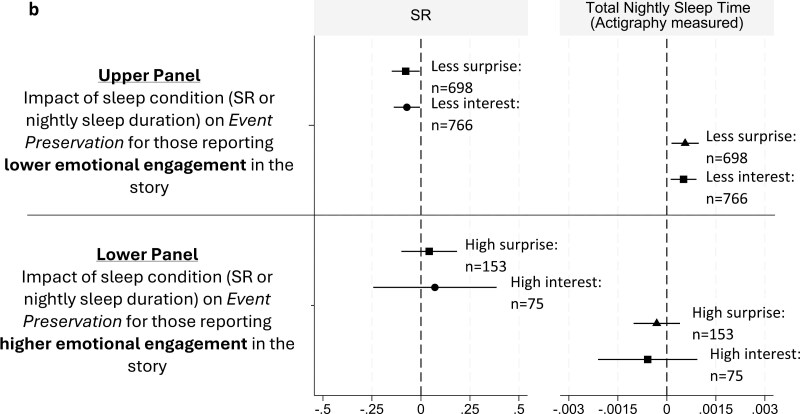
Individual-level SR effects as moderated by emotional engagement in the story: Exploratory Analysis of (A) Details Preserved and (B) Event Preservation. Plot shows the coefficient estimate with 95% two-tailed test confidence interval on what we consider an exploratory hypothesis of an emotional-engagement moderation of the SR effect. Coefficient plots represent the estimated impact of the SR condition (left-hand panel) or nightly average minutes slept on *Details Preserved* in the story retell, relative to the source story provided. For full estimation results, see Appendix Tables B6 and B7.

The affective state reports on *Surprise* and *Interest* given after each story retell allowed for an exploratory analysis of whether affective engagement moderates the reported SR effect. Here, we first split the data into subsets of those who reported higher versus lower *Surprise* or *Interest—*high affective engagement was consider 7–9 on the 9-point Likert rating scale used. Estimation results from the split samples are summarized with coefficient plots for *Details* and *Event Preservation* outcomes in Figures 4a and 4b (results in the split samples showed no differences for *Characters*), and so we relegate these to the full estimation results in Appendix B (Tables B5-B7). While the high engagement subsets of *Surprise* and *Interest* retells represent fewer observations, and therefore wider confidence intervals on the key coefficient estimates, it is nonetheless clear that the significant sleep effects reported above are restricted to the subset of *less* affectively engaged participants. This is most clear in the lower panel estimates focused on *Event Preservation*.

## Discussion

We presented novel evidence regarding how commonly experienced levels of insufficient sleep likely impact story retell fidelity. The narrative story reproduction task we examined represents a type of building-block task that can inform broader domains of communication that involves information transmission. Furthermore, how one retells a story can have future implications because subsequent memories can align with our own distorted retelling [29].

Overall, our results show that story retell fidelity is reduced by SR. Specifically, introducing a single SR participant into the chain of retells reduces both the length of a retell (i.e. *Characters* preserved) and the *Details* preserved within the retell. Interestingly, group level analyses showed that the reporting of the key event only deteriorates when a story is retold by three SR participants in a row. Additional analyses showed: (1) the SR effects are present within individuals, as well as at the group level, and SR is estimated to cause a significant deteriorization of a story’s key event in these multi-variate regressions; and (2) the SR effects may be driven by those with the least affective engagement in the story (i.e. *Surprise* and *Interest*).

Successful recall of stories in this study likely required both declarative memory encoding and recall, as well as working memory capacity (given the immediate recall demands). Few prior studies have examined the impact of sleep loss on the ability to encode and then recall stories, specifically (e.g. on Wechsler Memory Scale logical memory subtest or similar), and these have typically been conducted with older adults (e.g. [30]). However, data do show deficits in list learning after sleep deprivation [31] or habitually shorter sleep [32], as well as deficits in working memory capacity after both sleep deprivation [16] and sleep restriction [33]. Thus, our findings are consistent with the prior literature and expand that literature to show sleep restriction interferes with declarative encoding and recall, even when the material to be learned is within a more salient context of a story. Sleep loss also interferes with emotional processing and the encoding of emotional memories [34]. This may explain why some sleep restricted participants showed relatively low affective engagement with the stories. However, our finding that those with the greatest affective engagement in the stories showed the smallest sleep restriction effects is not consistent with the broader sleep and emotional memory literature.

Retelling stories occurs regularly in everyday life [35], and how different categories of details are recalled differs by age and gender [36]. Though results are mixed regarding sex differences in how sleep loss affects cognition, there are well-known sex-related differences in episodic memory of the sort that is relevant to story retelling [37]. If sleep pressure builds more quickly in women than men [38], or if women recover from sleep pressure more slowly [39], then insufficient sleep could produce differential impacts by sex on cognitive function relevant to story retelling [37]. Though not a focus of this present paper, our data did not show significant sex differences in how SR impacted story retelling (see Appendix B, Figure B1).^10^

Of course, our study has several limitations. We only studied visual narrative reproduction, which may differ in important ways from oral communication. Also, high stakes message relay likely involves tools to assist recall beyond memory alone (e.g. written or recorded records, etc.). However, because the task we studied avoided additional cognitive domains relevant to communication (e.g. face-to-face queues, social interaction, audio processing), which also suffer with short-term sleep loss (see Beattie et al. [40]), in this sense, our study can be considered a conservative assessment of how SR impacts serial information reproduction. Our particular focus on relatively mild sleep restriction, while commonplace, does not reflect the variety of levels or types of adverse sleep states experienced by many. By design, we aimed to exclude those with sleep disorders from the study, as we noted in our methods above, but we only obtained self-reports of whether one had a diagnosed (or suspected) sleep disorder. This is important, because the extent to which our sample participants may be unaware of having a sleep disorder represents a sleep-related confound in the data. Fortunately, the within-subjects nature of the study design implies that all participants were administered the story retell task under both SR and WR treatment conditions and therefore contribute to identifying a causal SR effect. However, if an underlying sleep disorder interacts with SR in a way that magnifies the SR effect on story retelling, then our findings would overestimate the pure effect of the sleep restrict condition. Finally, our participants were mostly college-aged young adults, which implies that additional research is needed to generalize our findings to other populations.

Our key findings highlight that, while serial (unaided) reproduction of information will result in some content loss, this effect is likely to be worse in households, workplaces, or contexts where those retelling the information/story suffer from insufficient sleep. An important exploratory finding showed that an emotional engagement with the story may provide resiliency against the detrimental effects of SR on story retell fidelity. Thus, emotional engagement when sharing important content has potential as a countermeasure against deleterious SR effects. That said, efforts to increase sleep or improve sleep hygiene offer a more direct solution to SR-related concerns in communication.

## Supplementary Material

zpaf026_suppl_Supplementary_Material

## Data Availability

The data and command files are available on the Open Science Framework at https://osf.io/p2teq/ (see OSF Storage folder = “Sleep restriction and story retelling” in the Files tab).

## Footnotes

1 The type of communication media is also relevant. For example, several studies have documented that recall from print and online stories (visual-only story) is similar and of higher fidelity than recall from radio (audio-only) or television (audio-visual) [18–21]. We focus on visual-only recall for this present study.

2 We note that a participant’s self-report of having “no diagnosed sleep disorder” and “no dietary restrictions” was not verified. Self-reported or suspected sleep disorder was asked in the separate screening survey used to generate the database of potential participants for the study (as was the screening instrument for diurnal preferences). Because “dietary restrictions” was not part of the original screening survey, the recruitment email noted that “no dietary restrictions” was a condition of the study.

3 Those enrolled were from a total of 3316 individuals who completed the required screening survey (administered each semester across six semesters at the first author’s academic institution). From those completing the screening survey, 1506 met the inclusion criteria and were included in the email invitations to participate.

4 In such an instance, the task would then confound the retelling with a moral choice environment where there exists a temptation for dishonesty, which has been found to increase when sleep restricted (see Dickinson and Masclet [28]).

5 Raters were not explicitly informed of participant condition (SR or WR) for a particular retell, but the alphanumeric subject coding (e.g. SRWR##, WRSR##) implied that an observant rater could infer this. The experimenter did not mention the participant condition to any of the raters, and none commented to the experimenter about the meaning of the anonymous participant coding.

6 We varied the listing order of the hypotheses here relative to our preregistration for ease of exposition.

7 All results were from one-sample *t*-tests of the treatment group outcome difference across weeks. For the control group participants, we found no significant differences across these measures comparing outcomes at the end of the first and the last WR treatment weeks (*p *> .05).

8 A few observations were lost due to a participant inadvertently advancing the screen in the online survey prior to reading the story, which participants reported to us. Regarding *Event Preservation*, we have fewer observations due to some retells having completely lost the key story event—we could not then calculate a decay rate for the subsequent retell.

9 The IPW correction is a weighted regression that accounted for sample selection in the number of participants originally enrolled in the study (*n* = 176) compared with those completing the study (*n* = 155: *n* = 118 Treatment, *n* = 37 Control)—the first-stage selection equation estimation results are shown in Appendix B, Table B4.

10 In our analysis, sex was defined by self-reported sex as assigned “at birth.”
