# Supplementary material for: The impact of insufficient sleep on the serial reproduction of information
Source: Sleep Adv. 2025 May 4;6(2):zpaf026. doi: 10.1093/sleepadvances/zpaf026 (PMC12146842; doi:10.1093/sleepadvances/zpaf026)
Supplement: zpaf026_suppl_Supplementary_Material [file zpaf026_suppl_supplementary_material.docx]

The impact of insufficient sleep on the serial reproduction of information

David L. Dickinson^1,2,3^ and Sean P.A. Drummond^4^

^1^ Appalachian State University, Economics and CERPA, Boone, NC, United States^

^2^ Economic Science Institute (ESI), Chapman University, Orange, CA, United States

^3^ Institute of Labor Economics (IZA), Bonn, Germany

^4^School of Psychological Sciences, Monash University, Clayton, Victoria, Australia^

^Work performance sites

Dual Corresponding Authors:

Sean P.A. Drummond

School of Psychological Sciences, Turner Institute for Brain and Mental Health

Monash University, 19 Innovation Wal, Room 542

Clayton, VIC 3800

Email: sean.drummond@monash.edu

David L. Dickinson

Economics Department, Appalachian State University, 416 Howard Street, Boone, NC 28608 USA

Email: dickinsondl@appstate.edu

**APPENDIX A: Pilot Data and Telephone Game task**

**FIGURE A1: Pilot data summary—similarity of original and novel stories**

**Notes:** n=20 unique participants retold each of the 6 story retellings, the original stories (Breithaupt et al., 2018) and the newly created Amanda, Jessica and Eric Stories. Graphs represent average scored outcomes from 2 independent scorings of each retold story.

**Original Stories** (details to score shaded, with key event in yellow)

**Jason story (yellow highlight is key event resolution detail) (24)**

- 1016 characters, 14 sentences, 24 details
  - Key problem: **lack of girlfriend/date**

Jason was a high school freshman.  He was not very outgoing, did not do well in sports and was a big fan of science fiction TV programs.  Near the beginning of the school year, he had an embarrassing moment playing football in gym class where the ball hit him in the head, causing his classmates to laugh at him and to tease him.  By the middle of the semester, the school dance was coming up and all of Jason's classmates were excited about it, but it did not interest Jason.  Later, his best friend found a date, and began pressuring Jason to go as well, telling him that it would be fun.  But Jason still remembered being laughed at by his whole class and did not want to get turned down.  Jason did not ask anyone out.  He stayed at home during the dance and avoided talking to his best friend about it.  A few weeks later, a girl, who was new to town, transferred into Jason's classes.  Jason found her attractive and liked her more and more as time went on.  But, he was too afraid to talk to her.  One day, he decided that to overcome his fear of talking to her he needed to assume an alternate identity.  He dressed up as a superhero and walked over to where she was sitting.  He mumbled that she looked nice and asked her if she would like to eat lunch with him.

**Sarah story (yellow highlight is key event resolution detail) (22)**

- 1045 characters, 13 sentences, 22 details
  - Key problem: **Emotional inability to face Mom**.

Sarah was a teenager living in a small town in the American Midwest.  She was a good student who did well in all of her classes, except history.  Her mother wanted her to do better in history and hired a student from the local college as a tutor.  On a day with exceptionally good weather, Sarah decided to spend her afternoon playing softball with her friends instead of attending her tutoring session.  The tutor called Sarah's mother and told her about Sarah's absence that afternoon.  The news embarrassed Sarah's mother, who scolded Sarah and lectured her on responsibility after she came home.  Sarah did not think her skipping tutoring was a big deal since her mother did not have to pay for the missed lesson, she had good grades in all her other classes, and she knew that her tutor was laidback and would not take her absence personally.  Sarah and her mother had a fight and Sarah ran out of the house.  She decided to go into the woods near her house.  In the woods, she made a fire and burnt everything her mother had ever given her.  That made her feel better and gave her the confidence to talk to her mother again.  Her mother had been waiting at home and was busying herself preparing dinner when Sarah got back.  Upon seeing her mother in the kitchen, Sarah apologized.

**Robert story (yellow highlight is key event resolution detail) (22)**

- 1102 characters, 15 sentences, 22 details
  - Key problem: **Don’t know exam answers**

Robert was a physics student attending a prestigious college on the East Coast.  Even though he wanted to become a scientist, the college's requirements demanded that he take at least one history class.  Robert decided to get the class out of the way in his freshman year.  He enrolled in the only one available, an American history class with a professor who had a reputation of being stern but knowledgeable.  Robert felt that the class was interesting enough and did well in the assignments and on the midterm exam.  Unfortunately for Robert, the history final exam was scheduled on the same day as the final for an important physics class.  The day before the exams, Robert stayed up half of the night, mostly studying physics.  After taking the physics test in the morning he felt that he had done well.  At noon he sat down in class to take his history exam.  He knew the exam would be difficult, but he was shocked to see how hard it was.  He may not have studied enough, but this exam was simply not fair and he started sweating.  With an hour left, he asked for a bathroom break and left the room.  In the bathroom, he did sprints in front of the stalls to get his brain going.  While running, he hit his head on a door, but instead of confusing him, it seemed to cause everything to make sense.  Then he returned to the testing room to complete the exam.

***********************************************************************************

**New Stories** (details to score shaded, with key event in yellow)

**Jessica story (yellow highlight is key event resolution detail) (22)**

- 1009 characters, 16 sentences, 22 details
  - Key problem: **distress about vacation time off from work**

Jessica was married and had 2 children, and her husband was a blue-collar factory worker. Jessica had a part-time job at a local coffee shop. Jessica needed to ask for 2 weeks off from her job for a family vacation. She was concerned that her boss would not allow that. Her boss was rather mean at times, and complained once when Jessica needed to request one week off. Jessica feared that she may be fired if she asked for 2 weeks off from work, but her husband told her not to worry. Jessica wanted to catch her boss in a good mood before asking for the time off. One day, her boss seemed in a really good mood, and so Jessica asked for the 2 weeks off. Her boss suddenly became upset and told Jessica to choose between her job and her vacation. Her boss’s reaction shocked Jessica. Even some customers in the coffee-shop seemed uncomfortable with such a reaction. Jessica dropped the topic and cried once she got home. Jessica’s husband again told her to not worry, because just last week he found a box of money that was buried in the back yard. The box had enough money in it so that Jessica did not have to worry about her coffee shop job. That information made Jessica feel better. She had a really good night of sleep that evening.

**Eric story (yellow highlight is key event resolution detail) (24)**

- 1066 characters, 14 sentences, 24 details
  - Key problem: **Farm preventing Eric’s piano dreams**

Eric was a teenager who grew up on the family farm. The farm had been in the family for several generations, Eric was an only child, and Eric was sure his father had planned on Eric taking over the farm one day. Eric enjoyed the farm, the animals, and working with his hands. However, he did not want the farm to become his life. Eric’s family had a piano in their house, and he spent many hours since childhood teaching himself how to play. Playing the piano seemed to come naturally to him. Eric was gifted at the piano, and he hoped to study music one day at a University. The summer before Eric’s senior year in high school, Eric’s father had a major heart attack and died. Eric’s whole family was devastated, and his dreams of studying music seemed to fade away as he was needed to help his mother on the farm in a full-time capacity. After a few weeks, Eric’s mother shared a letter written for Eric from his father. The letter told Eric that he and his mother saw Eric’s music talent and had saved enough money for him to go to college and pursue his dreams. Arrangements had been made to have Eric’s cousin help with the farm until it could be sold. Eric’s mother was ready to move on from farm life as well after her husband’s death. Eric went to college and eventually became a professional pianist.

**Amanda story (yellow highlight is key event resolution detail) (24)**

- 1222 characters, 15 sentences, 24 details
  - Key problem: **Concern over controlling new employee**

Amanda was a timid woman in her 20’s. She lived in a big city where life was fast-paced, but she loved to spend time in the mountains. Amanda had a pet cat in her studio apartment, and her social time was often spent with friends from her job at an insurance company. Her friends were also somewhat timid, and certainly would not be considered risk-takers. One day, the insurance company hired a new employee. After a few weeks, it became clear that this new employee had a leadership personality and was also a thrill-seeker. The new employee was an avid rock-climber, scuba-diver, and had gone sky-diving several times. Stories of this new employee’s adventures amazed Amanda’s timid friends. The new employee invited Amanda and her friends to go hiking at a place a couple hours from the city one weekend. Amanda was conflicted because her friends wanted to go, but she knew the hike location was dangerous due to high cliffs in that area. Amanda’s co-workers seemed hypnotized by this new employee’s charismatic personality. Sensing Amanda’s resistance, the new employee threatened to start a rumor that would get Amanda fired if she did not go on the hiking trip. Unfortunately for the new employee, this threat was caught on the company’s security camera. After Amanda shared this with her boss, the new employee was fired, and Amanda’s friends then realized they had been deceived. Afterwards, Amanda enjoyed a weekend hike with her friends at a safer location.

**************************************************************************************

**TELEPHONE GAME TASK INSTRUCTIONS**

R-E-Ja story block instructions

(dotted lines show page breaks in survey. Stories presented in randomized order)

-------------------------------------

Please input your experiment subject code (e.g., SRWR113, WRSR18, etc), *not your name,*in the space below.
(should be 4 letters followed by 1-3 numbers)

-------------------------------------

Please mark the number that best corresponds to how sleepy you feel right now. You may mark any number, but mark only one number.

- 1. Extremely alert
- 2.
- 3. Alert
- 4.
- 5. Neither alert nor sleepy
- 6.
- 7. Sleepy - but no difficulty remaining awake
- 8.
- 9. Extremely sleepy - fighting sleep

-------------------------------------

We are interested in understanding the impact of sleep on how people communicate with one another. In this study, we will show you a piece of text someone has written and give you time to read it. 
**Your task will be to read the text and try to recall as much of this information to the best of your ability.**You will do this by rewriting the story in your own words and then answering some relevant questions.
It is important that you understand the text, but please do not copy the story, take a picture of it, or use any assistance in doing this task. The task should take no more than 15-20 minutes to complete. 
 
Please begin.

-------------------------------------

Please spend sufficient time to fully read the following story. You will be asked to retell the story on a following screen.
 

 "Robert was a physics student attending a prestigious college on the East Coast. Even though he wanted to become a scientist, the college's requirements demanded that he take at least one history class. Robert decided to get the class out of the way in his freshman year. He enrolled in the only one available, an American history class with a professor who had a reputation of being stern but knowledgeable. Robert felt that the class was interesting enough and did well in the assignments and on the midterm exam. Unfortunately for Robert, the history final exam was scheduled on the same day as the final for an important physics class. The day before the exams, Robert stayed up half of the night, mostly studying physics. After taking the physics test in the morning he felt that he had done well. At noon he sat down in class to take his history exam. He knew the exam would be difficult, but he was shocked to see how hard it was. He may not have studied enough, but this exam was simply not fair and he started sweating. With an hour left, he asked for a bathroom break and left the room. In the bathroom, he did sprints in front of the stalls to get his brain going. While running, he hit his head on a door, but instead of confusing him, it seemed to cause everything to make sense. Then he returned to the testing room to complete the exam."

-------------------------------------

Please retell the story you just read on the previous page by typing the story into the text box below. Please attempt to retell the story as accurately as possible.

[TEXT BOX HERE]

-------------------------------------

Please tell us how interested you were in the story you just retold using the scale below.

|  | **Not interested  at all** | | | | **Medium level of interest** | | | | **Very interested** | | | |
| --- | --- | --- | --- | --- | --- | --- | --- | --- | --- | --- | --- | --- |
|  | | 1 | 2 | 3 | | 4 | 5 | 6 | | 7 | 8 | 9 |

| **How interested I was** in the story | 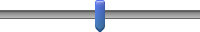 |
| --- | --- |

-------------------------------------

Please tell us how surprised you were in the story you just retold using the scale below.

|  | **Not surprised  at all** | | | | **Medium level of surprise** | | | | **Very surprised** | | | |
| --- | --- | --- | --- | --- | --- | --- | --- | --- | --- | --- | --- | --- |
|  | | 1 | 2 | 3 | | 4 | 5 | 6 | | 7 | 8 | 9 |

| **How surprised I was** in the story | 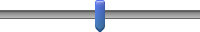 |
| --- | --- |

-------------------------------------

Please spend sufficient time to fully read the following story. You will be asked to retell the story on a following screen.
 
 "Eric was a teenager who grew up on the family farm. The farm had been in the family for several generations, Eric was an only child, and Eric was sure his father had planned on Eric taking over the farm one day. Eric enjoyed the farm, the animals, and working with his hands. However, he did not want the farm to become his life. Eric’s family had a piano in their house, and he spent many hours since childhood teaching himself how to play. Playing the piano seemed to come naturally to him. Eric was gifted at the piano, and he hoped to study music one day at a University. The summer before Eric’s senior year in high school, Eric’s father had a major heart attack and died. Eric’s whole family was devastated, and his dreams of studying music seemed to fade away as he was needed to help his mother on the farm in a full-time capacity. After a few weeks, Eric’s mother shared a letter written for Eric from his father. The letter told Eric that he and his mother saw Eric’s music talent and had saved enough money for him to go to college and pursue his dreams. Arrangements had been made to have Eric’s cousin help with the farm until it could be sold. Eric’s mother was ready to move on from farm life as well after her husband’s death. Eric went to college and eventually became a professional pianist."

-------------------------------------

Please retell the story you just read on the previous page by typing the story into the text box below. Please attempt to retell the story as accurately as possible.

[TEXT BOX HERE]

-------------------------------------

Please tell us how interested you were in the story you just retold using the scale below.

|  | **Not interested  at all** | | | | **Medium level of interest** | | | | **Very interested** | | | |
| --- | --- | --- | --- | --- | --- | --- | --- | --- | --- | --- | --- | --- |
|  | | 1 | 2 | 3 | | 4 | 5 | 6 | | 7 | 8 | 9 |

| **How interested I was** in the story | 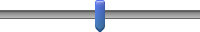 |
| --- | --- |

-------------------------------------

Please tell us how surprised you were in the story you just retold using the scale below.

|  | **Not surprised  at all** | | | | **Medium level of surprise** | | | | **Very surprised** | | | |
| --- | --- | --- | --- | --- | --- | --- | --- | --- | --- | --- | --- | --- |
|  | | 1 | 2 | 3 | | 4 | 5 | 6 | | 7 | 8 | 9 |

| **How surprised I was** in the story | 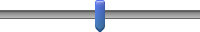 |
| --- | --- |

-------------------------------------

Please spend sufficient time to fully read the following story. You will be asked to retell the story on a following screen.
 
"Jessica was married and had 2 children, and her husband was a blue-collar factory worker. Jessica had a part-time job at a local coffee shop. Jessica needed to ask for 2 weeks off from her job for a family vacation. She was concerned that her boss would not allow that. Her boss was rather mean at times, and complained once when Jessica needed to request one week off. Jessica feared that she may be fired if she asked for 2 weeks off from work, but her husband told her not to worry. Jessica wanted to catch her boss in a good mood before asking for the time off. One day, her boss seemed in a really good mood, and so Jessica asked for the 2 weeks off. Her boss suddenly became upset and told Jessica to choose between her job and her vacation. Her boss’s reaction shocked Jessica. Even some customers in the coffee-shop seemed uncomfortable with such a reaction. Jessica dropped the topic and cried once she got home. Jessica’s husband again told her to not worry, because just last week he found a box of money that was buried in the back yard. The box had enough money in it so that Jessica did not have to worry about her coffee shop job. That information made Jessica feel better.  She had a really good night of sleep that evening."

-------------------------------------

Please retell the story you just read on the previous page by typing the story into the text box below. Please attempt to retell the story as accurately as possible.

[TEXT BOX HERE]

-------------------------------------

Please tell us how interested you were in the story you just retold using the scale below.

|  | **Not interested  at all** | | | | **Medium level of interest** | | | | **Very interested** | | | |
| --- | --- | --- | --- | --- | --- | --- | --- | --- | --- | --- | --- | --- |
|  | | 1 | 2 | 3 | | 4 | 5 | 6 | | 7 | 8 | 9 |

| **How interested I was** in the story | 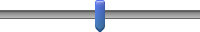 |
| --- | --- |

-------------------------------------

Please tell us how surprised you were in the story you just retold using the scale below.

|  | **Not surprised  at all** | | | | **Medium level of surprise** | | | | **Very surprised** | | | |
| --- | --- | --- | --- | --- | --- | --- | --- | --- | --- | --- | --- | --- |
|  | | 1 | 2 | 3 | | 4 | 5 | 6 | | 7 | 8 | 9 |

| **How surprised I was** in the story | 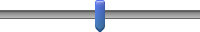 |
| --- | --- |

-------------------------------------

**APPENDIX B: Additional estimation results**

Note: The binary indicator variable, *Female*, is defined in our data as *Female* = 1 if participant responded in the screening survey to the question “What is your sex (assigned at birth)?” by indicating “Female”. Other response options were “Male”, and “Rather not say”, both of which would be coded as *Female* = 0.

| **TABLE B1: The impact of SR on Characters preserved** | | | |  |  |  |
| --- | --- | --- | --- | --- | --- | --- |
| Dependent Variable = *Characters Preserved* | Models using **binary SR indicator** variable to control for sleep state | | | Models using **continuous (actigraphy measured) nightly sleep** as control for sleep state | | |
| Independent Variables | (1) | (2) | (3) | (4) | (5) | (6) |
| SR (=1) | -0.02 | -0.02 | -0.01 |  |  |  |
|  | (0.01) | (0.01) | (0.02) |  |  |  |
| Avg Nightly Sleep (min) |  |  |  | 0.00 | 0.00 | 0.00 |
|  |  |  |  | (0.00) | (0.00) | (0.00) |
| Age |  | 0.01** | 0.01** |  | 0.01** | 0.01** |
|  |  | (0.00) | (0.00) |  | (0.00) | (0.00) |
| Female (=1) |  | 0.08** | 0.09** |  | 0.08** | 0.09** |
|  |  | (0.03) | (0.03) |  | (0.03) | (0.03) |
| Minority (=1) |  | 0.02 | 0.02 |  | 0.02 | 0.02 |
|  |  | (0.04) | (0.04) |  | (0.04) | (0.04) |
| MEQ |  | 0.01** | 0.01** |  | 0.01** | 0.01** |
|  |  | (0.00) | (0.00) |  | (0.00) | (0.00) |
| CRTscore |  | 0.02** | 0.02** |  | 0.02** | 0.02** |
|  |  | (0.01) | (0.01) |  | (0.01) | (0.01) |
| Repeat Administration (=1) |  | 0.01 | 0.01 |  | 0.01 | 0.01 |
|  |  | (0.01) | (0.01) |  | (0.01) | (0.01) |
| Constant | -0.25** | -0.75** | -0.75** | -0.32** | -0.80** | -0.77** |
|  | (0.01) | (0.08) | (0.09) | (0.05) | (0.09) | (0.10) |
| IPW Correction | NO | NO | YES | NO | NO | YES |
| Observations | 924 | 924 | 924 | 924 | 924 | 924 |
| # Participants | 155 | 155 | 155 | 155 | 155 | 155 |
| R-squared | 0.00165 | 0.118 | 0.12 | 0.00492 | 0.117 | 0.12 |

**p* < .05, ***p* < .01 for the 1-tailed test of the preregistered hypothesis tests on the SR or Avg Nightly Sleep Variables (all other significance based on 2-tailed tests). Random effects GLS regressions with robust standard errors (in parenthesis) clustered on participant. All models included a fixed effect variable for each different story. IPW corrected regressions (columns (3) and (6)) are linear ordinary least squares regressions (weights based on selection equation results shown in Table B4).

| **TABLE B2: The impact of SR on Details preserved** | | | |  |  |  |
| --- | --- | --- | --- | --- | --- | --- |
| Dependent Variable =  *Details Preserved* | Models using **binary SR indicator** variable to control for sleep state | | | Models using **continuous (actigraphy measured) nightly sleep** as control for sleep state | | |
| Independent Variables | (1) | (2) | (3) | (4) | (5) | (6) |
| SR (=1) | -0.02 | -0.02 | -0.02 |  |  |  |
|  | (0.01) | (0.01) | (0.01) |  |  |  |
| Avg Nightly Sleep (min) |  |  |  | 0.0002* | 0.000015* | 0.0002* |
|  |  |  |  | (0.0001) | (0.00008) | (0.0001) |
| Age |  | 0.01** | 0.01** |  | 0.01** | 0.01** |
|  |  | (0.00) | (0.00) |  | (0.00) | (0.00) |
| Female (=1) |  | 0.10** | 0.11** |  | 0.10** | 0.10** |
|  |  | (0.02) | (0.02) |  | (0.02) | (0.02) |
| Minority (=1) |  | 0.02 | 0.02 |  | 0.02 | 0.02 |
|  |  | (0.03) | (0.03) |  | (0.03) | (0.03) |
| MEQ |  | 0.01 | 0.01 |  | 0.01 | 0.01 |
|  |  | (0.00) | (0.00) |  | (0.00) | (0.00) |
| CRTscore |  | 0.02** | 0.02** |  | 0.02** | 0.02** |
|  |  | (0.01) | (0.01) |  | (0.01) | (0.01) |
| Repeat Administration (=1) |  | 0.01 | 0.01 |  | 0.01 | 0.01 |
|  |  | (0.01) | (0.01) |  | (0.01) | (0.01) |
| Constant | -0.23** | -0.62** | -0.63** | -0.31** | -0.68** | -0.70** |
|  | (0.01) | (0.08) | (0.08) | (0.04) | (0.08) | (0.09) |
| IPW CORRECTION | NO | NO | YES | NO | NO | YES |
| Observations | 924 | 924 | 924 | 924 | 924 | 924 |
| # Participants | 155 | 155 | 155 | 155 | 155 | 155 |
| R-squared | 0.00295 | 0.121 | 0.13 | 0.0176 | 0.124 | 0.13 |

**p* < .05, ***p* < .01 for the 1-tailed test of the preregistered hypothesis tests on the SR or Avg Nightly Sleep Variables (all other significance based on 2-tailed tests). Random effects GLS regressions with robust standard errors (in parenthesis) clustered on participant. All models included a fixed effect variable for each different story. IPW corrected regressions (columns (3) and (6)) are linear ordinary least squares regressions (weights based on selection equation results shown in Table B4).

| **TABLE B3: The impact of SR on Key Event Preservation** | | | | | | |
| --- | --- | --- | --- | --- | --- | --- |
| Dependent Variable =  *Event Preservation* | Models using **binary SR indicator** variable to control for sleep state | | | Models using **continuous (actigraphy measured) nightly sleep** as control for sleep state | | |
| Independent Variables | (1) | (2) | (3) | (4) | (5) | (6) |
| SR (=1) | -0.07* | -0.07* | -0.07* |  |  |  |
|  | (0.03) | (0.03) | (0.03) |  |  |  |
| Avg Nightly Sleep (min) |  |  |  | 0.0006** | 0.0005** | 0.0005* |
|  |  |  |  | (0.0002) | (0.0002) | (0.0002) |
| Age |  | 0.00 | 0.00 |  | 0.00 | 0.00 |
|  |  | (0.01) | (0.01) |  | (0.01) | (0.01) |
| Female (=1) |  | 0.09* | 0.09* |  | 0.08 | 0.08 |
|  |  | (0.04) | (0.04) |  | (0.04) | (0.04) |
| Minority (=1) |  | 0.06 | 0.06 |  | 0.06 | 0.06 |
|  |  | (0.06) | (0.06) |  | (0.07) | (0.06) |
| MEQ |  | 0.00 | 0.00 |  | 0.00 | -0.00 |
|  |  | (0.01) | (0.01) |  | (0.01) | (0.01) |
| CRTscore |  | 0.02 | 0.02 |  | 0.01 | 0.01 |
|  |  | (0.01) | (0.01) |  | (0.01) | (0.01) |
| Repeat Administration (=1) |  | -0.04 | -0.04 |  | -0.04 | -0.04 |
|  |  | (0.04) | (0.04) |  | (0.04) | (0.04) |
| Constant | -0.12** | -0.31 | -0.30 | -0.38** | -0.50** | -0.49** |
|  | (0.02) | (0.16) | (0.16) | (0.08) | (0.16) | (0.16) |
| IPW CORRECTION | NO | NO | YES | NO | NO | YES |
| Observations | 851 | 851 | 851 | 851 | 851 | 851 |
| # Participants | 155 | 155 | 155 | 155 | 155 | 155 |
| R-squared | 0.00423 | 0.0212 | 0.02 | 0.00650 | 0.0213 | 0.02 |

**p* < .05, ***p* < .01 for the 1-tailed test of the preregistered hypothesis tests on the SR or Avg Nightly Sleep Variables (all other significance based on 2-tailed tests). Random effects GLS regressions with robust standard errors (in parenthesis) clustered on participant. All models included a fixed effect variable for each different story. IPW corrected regressions (columns (3) and (6)) are linear ordinary least squares regressions (weights based on selection equation results shown in Table B4).

**TABLE B4**: Selection Equation Probit Results

Dep Var = 1 if completing the protocol conditional on enrollment

|  | (1) | (2) |
| --- | --- | --- |
| VARIABLES | coefficient | st. error |
| Female | 0.30 | (0.30) |
| Race | -0.08 | (0.41) |
| Age | 0.06 | (0.07) |
| LWselfTST | -0.14 | (0.18) |
| LNselfTST | -0.02 | (0.11) |
| OptSleep | 0.24 | (0.16) |
| Depression | -0.12 | (0.16) |
| Anxiety | 0.02 | (0.06) |
| Epworth | -0.12 | (0.05)** |
| MEQ | 0.02 | (0.05) |
| control | 0.58 | (0.42) |
| Constant | 0.27 | (2.76) |
| Observations | 175 |  |
| Chi-squared | 15.30 |  |
| Pseudo-Rsquared | 0.123 |  |

Standard errors in parentheses

** p<0.01, * p<0.05

**Notes**: n=176 participants enrolled in the study

while 155 completed the study.

One participant was omitted due to incomplete data from online screening survey.

A *Race* categories variable used in place of indicator for *Minority* (= 1 if Hispanic and/or non-Caucasian) perfectly predicts completing the protocol--i.e.

the few minority participants all completed the study

| **TABLE B5: The impact of SR on Characters by level of affective engagement**  **Dep Variable: *CHARACTERS* preserved in retell (relative to source story)** | | | | | | | | |
| --- | --- | --- | --- | --- | --- | --- | --- | --- |
|  | (1) | (2) | (3) | (4) | (5) | (6) | (7) | (8) |
| VARIABLES | Lower Surprise | High Surprise | Lower Interest | High Interest | Lower Surprise | High Surprise | Lower Interest | High Interest |
| SR (=1) | -0.01 | -0.02 | -0.02 | 0.04 | --- | --- | --- | --- |
|  | (0.01) | (0.04) | (0.01) | (0.06) |  |  |  |  |
| Avg Nightly Sleep (min) | --- | --- | --- | --- | 0.000 | -0.000 | 0.000 | -0.000 |
|  |  |  |  |  | (0.000) | (0.000) | (0.000) | (0.000) |
| Age | 0.01** | 0.01* | 0.01** | 0.03* | 0.01** | 0.01* | 0.01** | 0.03* |
|  | (0.00) | (0.01) | (0.00) | (0.01) | (0.00) | (0.01) | (0.00) | (0.01) |
| Female (=1) | 0.07* | 0.11** | 0.08** | 0.15** | 0.07* | 0.11** | 0.07* | 0.15** |
|  | (0.03) | (0.03) | (0.03) | (0.05) | (0.03) | (0.03) | (0.03) | (0.05) |
| Minority (=1) | 0.02 | 0.01 | 0.01 | -0.03 | 0.02 | 0.01 | 0.01 | -0.03 |
|  | (0.04) | (0.05) | (0.04) | (0.06) | (0.04) | (0.04) | (0.04) | (0.06) |
| MEQ | 0.01** | 0.00 | 0.01** | 0.01 | 0.01** | 0.00 | 0.01** | 0.01 |
|  | (0.00) | (0.01) | (0.00) | (0.01) | (0.00) | (0.01) | (0.00) | (0.01) |
| CRTscore | 0.02** | 0.02* | 0.02** | 0.01 | 0.02** | 0.02* | 0.02** | 0.01 |
|  | (0.01) | (0.01) | (0.01) | (0.01) | (0.01) | (0.01) | (0.01) | (0.01) |
| Repeat Administration (=1) | 0.01 | -0.02 | 0.00 | 0.02 | 0.01 | -0.02 | 0.00 | 0.02 |
|  | (0.01) | (0.03) | (0.01) | (0.06) | (0.01) | (0.03) | (0.01) | (0.06) |
| Constant | -0.78** | -0.63** | -0.75** | -1.00** | -0.80** | -0.59** | -0.81** | -0.79* |
|  | (0.08) | (0.13) | (0.09) | (0.28) | (0.09) | (0.14) | (0.09) | (0.36) |
| Observations | 766 | 158 | 845 | 79 | 766 | 158 | 845 | 79 |
| # Participants | 155 | 86 | 153 | 46 | 155 | 86 | 153 | 46 |
| R-squared | 0.121 | 0.171 | 0.116 | 0.264 | 0.121 | 0.183 | 0.114 | 0.269 |

**p* < .05, ***p* < .01 for the 2-tailed test of the exploratory hypotheses. Random effects GLS regressions with robust standard errors (in parenthesis) clustered on participant. All models included a fixed effect variable for each different story.

| **TABLE B6: The impact of SR on Details by level of affective engagement**  **Dep Variable: *DETAILS* preserved in retell (relative to source story)** | | | | | | | | |
| --- | --- | --- | --- | --- | --- | --- | --- | --- |
|  | (1) | (2) | (3) | (4) | (5) | (6) | (7) | (8) |
| VARIABLES | Lower Surprise | High Surprise | Lower Interest | High Interest | Lower Surprise | High Surprise | Lower Interest | High Interest |
| SR (=1) | -0.01 | 0.005 | -0.02 | 0.07 | --- | --- | --- | --- |
|  | (0.01) | (0.03) | (0.01) | (0.05) |  |  |  |  |
| Avg Nightly Sleep (min) | --- | --- | --- | --- | 0.0001 | -0.0001 | 0.0002* | -0.0005 |
|  |  |  |  |  | (0.0001) | (0.0002) | (0.0001) | (0.0003) |
| Age | 0.01** | 0.01 | 0.01** | 0.03* | 0.01** | 0.01 | 0.01** | 0.02* |
|  | (0.00) | (0.00) | (0.00) | (0.01) | (0.00) | (0.00) | (0.00) | (0.01) |
| Female (=1) | 0.10** | 0.10** | 0.10** | 0.13** | 0.10** | 0.10** | 0.09** | 0.14** |
|  | (0.02) | (0.03) | (0.02) | (0.04) | (0.02) | (0.03) | (0.02) | (0.04) |
| Minority (=1) | 0.01 | 0.03 | 0.01 | -0.00 | 0.01 | 0.03 | 0.01 | -0.01 |
|  | (0.03) | (0.04) | (0.03) | (0.04) | (0.03) | (0.04) | (0.03) | (0.04) |
| MEQ | 0.01 | 0.00 | 0.01 | 0.01 | 0.01 | 0.00 | 0.01 | 0.01 |
|  | (0.00) | (0.00) | (0.00) | (0.01) | (0.00) | (0.00) | (0.00) | (0.01) |
| CRTscore | 0.02** | 0.03** | 0.02** | 0.02* | 0.02** | 0.03** | 0.02** | 0.02* |
|  | (0.01) | (0.01) | (0.01) | (0.01) | (0.01) | (0.01) | (0.01) | (0.01) |
| Repeat Administration (=1) | 0.02 | -0.01 | 0.01 | 0.03 | 0.02 | -0.01 | 0.01 | 0.03 |
|  | (0.01) | (0.02) | (0.01) | (0.05) | (0.01) | (0.02) | (0.01) | (0.05) |
| Constant | -0.64** | -0.50** | -0.63** | -0.86** | -0.69** | -0.44** | -0.70** | -0.57* |
|  | (0.08) | (0.10) | (0.08) | (0.20) | (0.09) | (0.10) | (0.08) | (0.29) |
| Observations | 766 | 158 | 845 | 79 | 766 | 158 | 845 | 79 |
| # Participants | 155 | 86 | 153 | 46 | 155 | 86 | 153 | 46 |
| R-squared | 0.112 | 0.235 | 0.118 | 0.327 | 0.116 | 0.251 | 0.122 | 0.336 |

**p* < .05, ***p* < .01 for the 2-tailed test of the exploratory hypotheses. Random effects GLS regressions with robust standard errors (in parenthesis) clustered on participant. All models included a fixed effect variable for each different story.

| **TABLE B7: The impact of SR on Event Preservation by level of affective engagement**  **Dep Variable: *EVENT PRESERVATION* maintained in retell (relative to source story)** | | | | | | | | |
| --- | --- | --- | --- | --- | --- | --- | --- | --- |
|  | (1) | (2) | (3) | (4) | (5) | (6) | (7) | (8) |
| VARIABLES | Lower Surprise | High Surprise | Lower Interest | High Interest | Lower Surprise | High Surprise | Lower Interest | High Interest |
| SR (=1) | -0.08* | 0.04 | -0.07* | 0.07 | --- | --- | --- | --- |
|  | (0.04) | (0.07) | (0.03) | (0.16) |  |  |  |  |
| Avg Nightly Sleep (min) | --- | --- | --- | --- | 0.001** | -0.000 | 0.001** | -0.001 |
|  |  |  |  |  | (0.0002) | (0.000) | (0.0002) | (0.001) |
| Age | 0.01 | -0.00 | 0.00 | 0.05* | 0.01 | -0.00 | 0.00 | 0.04* |
|  | (0.01) | (0.01) | (0.01) | (0.02) | (0.01) | (0.01) | (0.01) | (0.02) |
| Female (=1) | 0.09 | 0.12 | 0.10* | 0.04 | 0.08 | 0.12 | 0.09* | 0.06 |
|  | (0.05) | (0.07) | (0.05) | (0.06) | (0.05) | (0.07) | (0.05) | (0.06) |
| Minority (=1) | 0.04 | 0.16 | 0.09 | -0.06 | 0.05 | 0.16 | 0.09 | -0.06 |
|  | (0.07) | (0.11) | (0.07) | (0.07) | (0.07) | (0.11) | (0.07) | (0.07) |
| MEQ | -0.00 | 0.01 | 0.00 | 0.00 | -0.00 | 0.01 | -0.00 | 0.00 |
|  | (0.01) | (0.01) | (0.01) | (0.01) | (0.01) | (0.01) | (0.01) | (0.01) |
| CRTscore | 0.02 | 0.02 | 0.02 | -0.02 | 0.01 | 0.02 | 0.02 | -0.02 |
|  | (0.01) | (0.02) | (0.01) | (0.02) | (0.01) | (0.02) | (0.01) | (0.02) |
| Repeat Administration (=1) | -0.03 | -0.10 | -0.05 | 0.05 | -0.03 | -0.10 | -0.05 | 0.05 |
|  | (0.04) | (0.07) | (0.04) | (0.16) | (0.04) | (0.07) | (0.04) | (0.15) |
| Constant | -0.34 | -0.32 | -0.29 | -1.06** | -0.56** | -0.19 | -0.50** | -0.73 |
|  | (0.18) | (0.24) | (0.17) | (0.34) | (0.17) | (0.30) | (0.17) | (0.46) |
| Observations | 698 | 153 | 776 | 75 | 698 | 153 | 776 | 75 |
| # Participants | 155 | 84 | 153 | 45 | 155 | 84 | 153 | 45 |
| R-Squared | 0.0205 | 0.109 | 0.0268 | 0.108 | 0.0214 | 0.110 | 0.0272 | 0.112 |

**p* < .05, ***p* < .01 for the 2-tailed test of the exploratory hypotheses. Random effects GLS regressions with robust standard errors (in parenthesis) clustered on participant. All models included a fixed effect variable for each different story.

**FIGURE B1:** Analysis of individual effects by sex

**Notes:** Plot shows the coefficient estimate with the 99% (thin line) and 95% (thick line) 1-tail test confidence interval on the preregistered hypothesis (for comparison with pooled results in Figure 2 of main text).
